# Neuroinflammation as a Link in Parkinson’s and Alzheimer’s Diseases: A Systematic Review and Meta-Analysis

**DOI:** 10.14336/AD.2024.1174

**Published:** 2025-12-07

**Authors:** Anna Tylutka, Piotr Żabiński, Łukasz Walas, Agnieszka Zembron-Lacny

**Affiliations:** ^1^Department of Applied and Clinical Physiology, Collegium Medicum University of Zielona Gora, Zielona Gora, Poland.; ^2^Student Research Group, University of Zielona Gora, Collegium Medicum University of Zielona Gora, Zielona Gora, Poland.; ^3^Institute of Dendrology, Polish Academy of Sciences, Kórnik, Poland.

**Keywords:** cytokines, Alzheimer's disease, Parkinson disease, neuroinflammation

## Abstract

Neuroinflammation plays a critical role in Alzheimer's (AD) and Parkinson's diseases (PD) onset, pathophysiology, and progression. The aim of our meta-analysis was to review the available literature to assess the role of neuroinflammation in the pathogenesis of the two most common neurological diseases: Parkinson's disease and Alzheimer's disease. Two medical databases were searched: Web of Science and PubMed in the period from 2009-2023, where a total of 37 publications that met the inclusion criteria were selected for further evaluation. Both patients with AD and with PD showed statistically significantly higher levels of interleukin IL-6 compared to the control group: *p*-value of 0.0034 for AD (SMD, 1.17; 95% CI, 0.39-1.96) and *p*-value of 0.0487 for PD (SMD 0.29 95% Cl 0.00-0.59). In AD patients, statistical significance (for random effect) was also observed for IL-1β, where higher values of this cytokine were recorded in patients compared to controls *(p*-value <0.001). In turn, in patients with PD, apart from IL-6, statistical significance was also observed for tumor necrosis factor-α (TNF-α) (*p*= 0.0431, SMD 0.52 95%Cl 0.02-1.02). Significant heterogeneity was also recorded (Q =85.48; P < 0.01; I^2^ = 87%). In both study groups, significant differences in common effect were observed for the anti-inflammatory cytokine IL-10, which could suggest a protective effect of this cytokine in patients with neurodegenerative diseases. The obtained results reinforce the existing clinical evidence that Alzheimer's and Parkinson's diseases are accompanied by an inflammatory response, with considerably higher blood levels observed for pro-inflammatory cytokines: IL-6, TNF-α and IL-1β.

## INTRODUCTION

Demographic changes observed in society pose new challenges including the increasing incidence of neurodegenerative diseases such as Parkinson's disease (PD) and Alzheimer's disease (AD) in particular [1]. Both AD and PD share a specific pathohistological feature i.e. brain plaques. The development of brain plaques is a common pathohistological feature in both AD and PD. The difference between those two diseases, in that aspect, is that in AD, β-amyloid (Aβ) and tau proteins are misfolded and aggregated [2] while in PD the accumulation process relates to α-synuclein and tau proteins [3]. It has been proven *in vitro* that certain enzymes like neprilysin, insulin-degrading enzyme and matrix metalloproteinase 9 have the ability to degrade Aβ [4]. Not only are microglia thought to be able to phagocytose Aβ, but also to be one of the sources of insulin-degrading enzyme [5], neprilysin, and matrix metalloproteinase 9 [6]. Those findings lead to suspicion that microglia may play a role in degrading Aβ in humans. However, only soluble Aβ can be degraded in this manner since Aβ fibrillars are mostly resistant to degradation. Moreover, Aβ degradation by the microglia is insufficient as the overproduction of cytokines downregulates the expression of Aβ phagocytosis receptors. Additionally, the Aβ-affected microglia may reduce the expression of neprilysin and insulin-degrading enzyme [7]. The available data also suggest that neuronal loss in such areas as media temporal lobe, praticulary hippocampus, correlates with symptoms of cognitive impairment in AD, as the region is crucial for memory formation and retrieval [8]. In PD the neuronal loss is primarily seen in dopamine neurons of the substantia nigra [9, 10]. According to Zhu et al. [11], and Collins et al. [12] the activation of microglial and the subsequent neuroinflammatory response play a crucial role in the loss of dopaminergic neurons in PD as these neurons exhibit heightened vulnerability to inflammatory processes.

Neuroinflammation occurs in all neurological diseases including the neurodegenerative ones [13]. Microglia and astrocytes are potentially the main sources of cytokines in AD. Although studies evaluating the role of pro- and anti-inflammatory cytokines in the pathogenesis of AD are still under investigation, there are reports in the literature that suggest that pro-inflammatory IL-6 may contribute to the progression of AD by interfering with glucose metabolism. De Felice et al. [14] describe that IL-6, upon binding to its receptor, triggers activation of the PI3K/Akt signaling pathway, which results in the activation of mTOR and the inhibition of PTEN (phosphatase and tensin homolog deleted on chromosome ten). Then, activated mTOR may affect the functioning of insulin receptor substrate 1 (IRS1), and consequently lead to impaired insulin sensitivity and glucose uptake. As regards TNF-α, activation TNF-α/TNFR1 signaling pathway is responsible for promoting neuronal necroptosis, which significantly contributes to the development of AD [15]. In regard to of anti-inflammatory cytokines, IL-2 is the most important. IL-2 exposure has been shown to activate Treg cells, which controls the development of autoimmune disease [16], and animal studies (mice) additionally show that IL-2 therapy also restores memory impairment and spinal cord density, reducing amyloid burden and amyloid plaque deposition [17]. Research by Patel et al. [18] on aging transgenic mice models of AD (TgAPPsw and PSAPP) demonstrated that an elevated Aβ level was responsible for enhanced activation of pro-inflammatory cytokines including tumor necrosis factor-α (TNF-α), interleukin (IL)-6, IL-1α and granulocyte-macrophage colony-stimulating factor (GM-CSF). Moreover, the exposure of microglia to pre-aggregated Aβ1-42 was shown to increase the production of pro-inflammatory cytokines (i.e., pro-IL-1β, IL-6, TNF-α), macrophage inflammatory peptide (MIP-1α) and macrophage colony-stimulating factor (M-CSF) [8]. The activation of caspase-1, which is necessary for IL-1β activation, was reported to be significantly increased in patients with diagnosed AD [19], and high levels of this cytokines were detected in microglia surrounding Aβ plaques in the brains of AD patients. Anti-inflammatory cytokines, such as IL-4 and IL-10, may exert neuroprotective effects and their high levels were observed to lower the risk of both AD and PD [20, 21]. The concentration of IL-8 was also observed to be connected with AD biomarkers, such as Aβ and tau, in AD patients, however, the available studies show contradictory findings. Both elevated [22] and lower [23] levels of IL-8 were recorded in the AD group, which suggests its limited diagnostic usefulness in this condition.

Numerous studies indicate that high levels of chronic inflammation led to increased oxidative stress in patients with PD [24]. Activated microglia secrete neurotoxic mediators and thus increase oxidative stress, which results in protein modification and misfolding of α-syn. Alpha-syn secreted and/or released into the extraneuronal environment after cell damage or death and activates surrounding microglia to perpetuate chronic inflammation. Indeed, excessive microglial activation significantly increases the expression of proinflammatory cytokines: IL-6, TNF-α and IL-1β, which contribute to the degeneration of dopaminergic neurons of the substantia nigra [25]. Activated microglia in PD patients secrete a number of pro-inflammatory cytokines, including TNF-α, IL-1β and IL-6, and their level assessment both in cerebrospinal fluid (CSF) and blood may prove to be a valuable diagnostic tool [26, 27]. The diagnostic usefulness of TNF-α level measurement in patients with PD was confirmed in the study conducted by Fu et al. [26] where the area under the ROC curve (AUC) value for this cytokine was recorded at 0.719 (p < 0.05, 95% CI: 0.655-0.784). Numerous studies demonstrated a significant increase in IL-6 - a cytokine that plays a key role in neuronal differentiation in PD patients [28, 29], however, some other reports failed to demonstrate this significance [30-32]. With regard to IL-8, the research findings on the role it plays in PD remain inconsistent. William Gray et al. [32] reported no significant differences in IL-8 levels between PD patients and the control group whereas IL-8 level in the study by Gupta et al. [33] found that IL-8 level was additionally correlated with the duration of the disease. Studies performed on animals or cell cultures recorded the neuroprotective effect that IL-10 produced on neuronal cells with glutamate [33] and/or lipopolysaccharide [34], but to date, studies of both IL-10 and IL-12 in PD patients have provided conflicting evidence and further research is required.

The levels of cytokines measured in serum or CSF may serve as early indicators of neuroinflammation and, consequently, neurodegeneration. Early diagnosis of AD or PD is crucial for adequate care to be provided for the patients [35, 36]. Our previous meta-analysis [37] demonstrated that monitoring proinflammatory cytokine levels (IL-6, TNF-α and IL-1β) could provide an insight into age-related diseases progression, enable the assessment of inflammation severity, thereby creating a valuable tool not only in clinical trials but also in everyday clinical practice. Therefore, the aim of this meta-analysis was to review the literature to assess the role of neuroinflammation in the pathogenesis of the two most common neurological diseases: Parkinson's disease and Alzheimer's disease

## MATERIALS AND METHODS

### Search strategy

The literature review and meta-analysis were designed in accordance with the applicable Preferred Reporting Items for Systematic Reviews and Meta-Analytics (PRISMA) 2021 guidelines [38]. The Web of Science and PubMed databases were searched for studies that investigated neuroinflammation (expressed levels of pro- and anti-inflammatory cytokines) in the two most common neurodegenerative diseases: Alzheimer's and Parkinson's diseases. The queries were last updated on 21 March 2024, and the full search strategy is presented in Table 1. The search code is available in the supplementary materials as the file titled *Search code.* The research protocol was registered in PROSPERO under the number: CRD42024502450.

**Table 1:** Search Strategy for PubMed database

Search #	Search Strategy	Items Found
**1**	cytokines	994,373
**2**	Parkinson	177,107
**3**	Alzheimer	229,813
**4**	(#2) OR (#3)	380,372
**5**	blood	5,586,845
**6**	therapy	11,502,922
**7**	(#1) AND (#4)	10,582
**8**	(#5) AND (#7)	2,642
**9**	(#8) NOT (#6)	1,664
**Filters**		
	Free full text	825
	English	816
	Humans	527

### Inclusion and Exclusion Criteria

The inclusion criteria were as follows: (a) the original studies had to include patients with confirmed Parkinson's disease and/or Alzheimer's disease, (b) blood serum or plasma was the material used to assess the level of cytokines, (c) cytokine levels were quantified and all units were standardized to pg/ml, (d) the studies had to include a control group in which the same cytokines were measured (e) data were presented mean values (SD [standard deviation]). The results whose data were presented in the form of the median (IQR [interquartile range]) or median (range) were standardized according to the formulas by Luo et al. [39] for the mean value and Wan et al. [40] for standard deviation. Reviewers independently evaluated the eligibility of the research papers found, and any disagreement was resolved through discussion. The exclusion criteria included: (a) animal studies, (b) *in vivo* studies, (c) studies in a language other than English (d) lack of completely raw data enabling full interpretation of the results (e) reviews, meta-analyses, protocols, editorials, letters, preprints whose full texts were unavailable (f) duplicate publications (g) studies in which the level of cytokines was assessed in biological material other than blood, (h) the cytokines the data on which were found in less than 3 records.

### Data extraction

The characteristics of the included literature comprised: the name of the first author, the year of publication, the number of people included in the study and the control groups, as well as sex (% women). All results were presented as means and standard deviations. All the collected data and any controversies were reviewed and resolved by a third author.

### Statistical Analysis

The 'meta' package in the R environment [41,42] was used to perform the statistical analyses. Heterogeneity between studies was tested using the 'metacont' function with the Standardized Mean Difference (SMD) method. The meta-analytical approach used inverse variance, restricted maximum-likelihood estimator for tau^2^, Q-Profile method for confidence interval of tau^2^ and tau, and Hedges’ g. Forrest plots were used to visualize heterogeneity among studies. Linear regression test of funnel plot asymmetry was prepared according to Egger et al. [43]. Funnel plots were used to assess publication bias. The significance threshold for all statistical tests was set at *p* < 0.05.

## RESULTS

### Study Search and Characteristics

**Figure 1.**
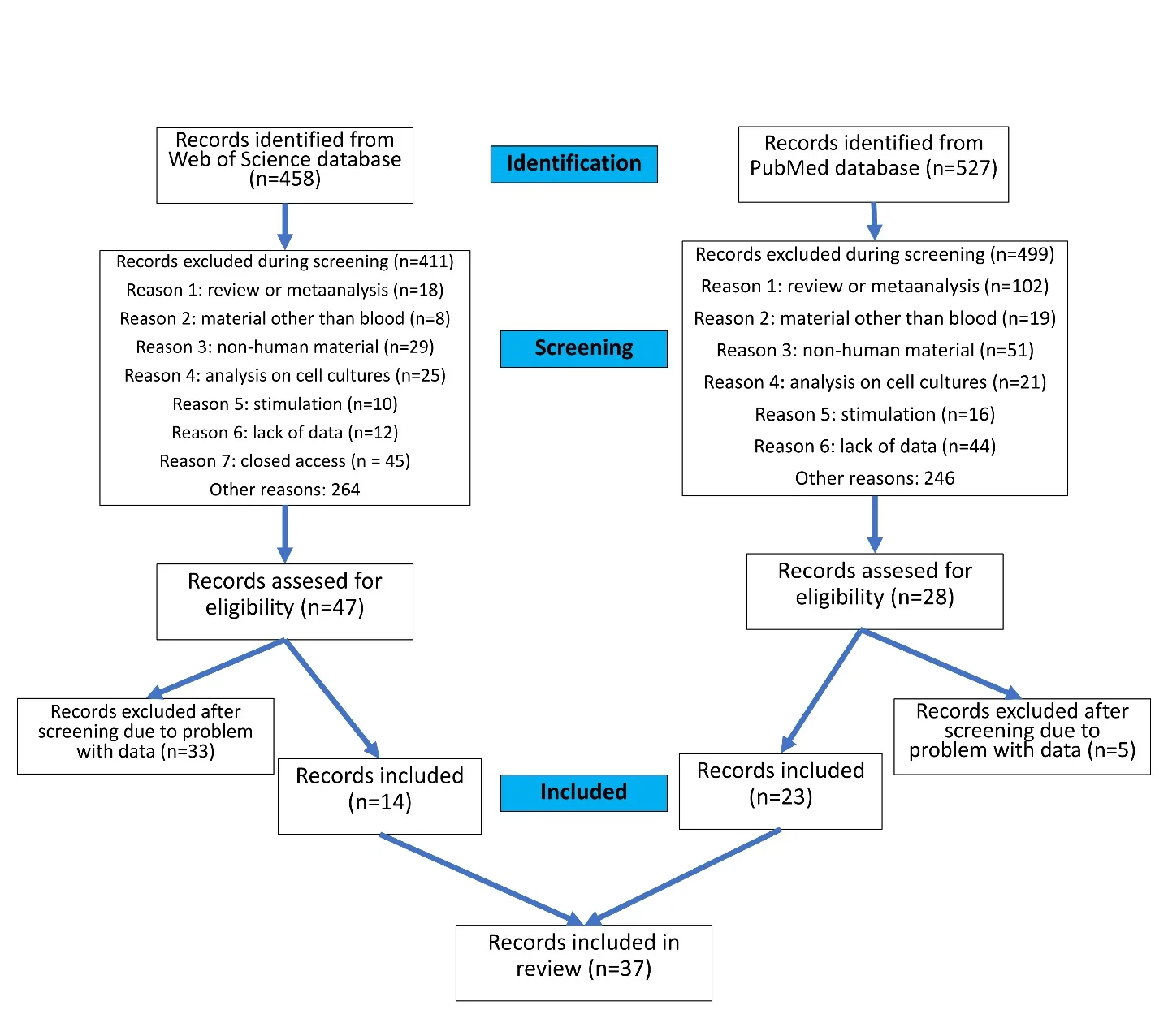
Preferred Reporting Items for Systematic Reviews and Meta-Analyses (PRISMA) flow diagram showing the paper selection process for inclusion.

After searching two databases: PubMed and Web of Science, 985 records were initially found, and after verification, the meta-analysis included 37 studies that assessed the level of pro- and anti-inflammatory cytokines in the blood of patients diagnosed with Alzheimer's and Parkinson's disease. After thorough verification of publications from the Web of Science, publications were excluded from further stages, among others, for the following reasons: analysis of cell culture (*n*=25), non-human material (*n*=29) and lack of data (*n*=12) etc. Consistently, the same criteria were applied to PubMed publications and excluded: *n*=21 reports on cell culture analyses, *n*= 51 investigations of non-human material and *n*=44 studies lacking in data (Fig. 1).

Publication time of the included studies ranged from 2009 to 2023. In the meta-analysis, the same cytokine panel was compared in both diseases and the following cytokines were included: pro-inflammatory cytokines: IL-6, IL-8, IL-12, IL-18, IL-1β, TNF-α and interferon-gamma (IFN-γ) and anti-inflammatory ones: IL-4, IL-10, IL-13. All cytokines were measured in either blood serum or blood plasma. Cytokine levels were expressed as mean and standard deviation in pg/mL units. The exact values are presented in the supplement in the tables: Supplementary 1 - values for AD, Supplementary 2 - values for PD. The total number of publications that assessed/measured cytokine levels in patients diagnosed with AD amounted to 25 papers (Table 2). The level of IL-6 was measured in as many as 17 publications. Since the publication by Eriksson et al. [44] involved two study groups i.e., incident and prevalent AD, the report was used in the analysis twice. Similarly, Li et al. [45] were used three times in our analyses as three independent study groups were investigated: mild, moderate and severe Alzheimer’s disease. In turn, the level of TNF-α was assessed 15 times and the research conducted by Li et al. [45] was analyzed three times. IL-1β was assessed 11 times and, as was the case with reports on TNF-α, the research by Li et al. [45] was analyzed three times. The lowest number of studie*s* (*n*=3) focused on the role of IL-4, IL-12 and IFN-γ in the pathogenesis of Alzheimer's disease.

**Table 2:** The list of publications included in this meta-analysis on Alzheimer’s disease.

Study	Cytokines									
	IL-1β	IL-4	IL-6	IL-8	IL-10	IL-12	IL-13	IL-18	IFN-γ	TNF-α
**Alsadany et al. [46]**				*						
**Amin et al. [47]**	*	*	*	*	*	*	*		*	*
**Björkqvist et al. [48]**				*						*
**Deniz et al. [49]**			*		*					*
**Erhardt et al. [50]**							*			
**Eriksson et al. [44]**			*							
**Galgani et al. [51]**	*		*		*	*				*
**Gu et al. [52]**			*							
**Huang et al. [53]**			*							*
**Italiani et al. [54]**	*							*		
**Jabbari Azad et al. [55]**		*	*						*	
**Leung et al. [56]**	*	*	*	*	*	*	*		*	*
**Li et al. [45]**	*		*							*
**Liang et al. [57]**	*									
**Park et al. [58]**	*									
**Raha et al. [59]**			*							
**Reale et al. [60]**								*		
**Scarabino et al. [61]**	*							*		
**Startin et al. [62]**	*		*		*					*
**Stoeck et al. [63]**			*				*			*
**Sun et al. [64]**	*		*	*						*
**Vida et al. [65]**			*							*
**Villareal et al. [66]**	*		*		*			*		*
**Wang et al. [67]**	*		*	*						*
**Wu et al. [68]**			*					*		

interleukin 1β (IL-1β); interleukin 4 (IL-4); interleukin 6 (IL-6); interleukin 8 (IL-8); interleukin 10 (IL-10); interleukin 12 (IL-12); interleukin 13 (IL-13); interleukin 18 (IL-18); interfon γ (IFN-γ); tumore necrosis factor α (TNF-α)

The total number of papers included in the meta-analysis for Parkinson's disease was *n*=13. As in the literature on Alzheimer's disease, most publications on Parkinson's disease assessed the level of IL-6 (9 publications). The investigation by Ton et al. [69] was included twice because the researchers analyzed two study groups: incident PD and prevalent PD. The study by Brockmann et al. [70] was included four times due to the nature of the results presented which the authors broke down by sex. The least attention was paid to the assessment of IL-12, 13 and INF-γ in the pathogenesis of Parkinson's disease (only 3 reports) (Table 3).

**Table 3:** The list of publications included in this meta-analysis in Parkinson’s diseases.

Study	Cytokines									
	IL-1β	IL-4	IL-6	IL-8	IL-10	IL-12	IL-13	IL-18	IFN-γ	TNF-α
**Adams et al. [71]**	*	*	*	*	*		*		*	*
**Björkqvist et al. [48]**				*						*
**Brockmann et al. [70]**	*	*	*	*	*			*		*
**Csencsits-Smith et al. [72]**	*	*	*			*	*		*	*
**Fan et al. [73]**	*									
**Ghit and Deeb [74]**					*	*				*
**Green et al. [30]**			*							*
**Hofmann et al. [75]**			*							
**Rentzos et al. [76]**					*	*				
**Tang et al. [77]**			*							
**Ton et al. [69]**			*							
**Williams-Gray et al. [32]**	*	*	*	*	*		*		*	*
**Xu et al. [78]**	*		*		*					*

interleukin 1β (IL-1β); interleukin 4 (IL-4); interleukin 6 (IL-6); interleukin 8 (IL-8); interleukin 10 (IL-10); interleukin 12 (IL-12); interleukin 13 (IL-13); interleukin 18 (IL-18); interfon γ (IFN-γ); tumore necrosis factor α (TNF-α)

### Assessment of pro- and anti-inflammatory cytokines in Alzheimer's disease

### Proinflammatory cytokines

**Figure 2.**
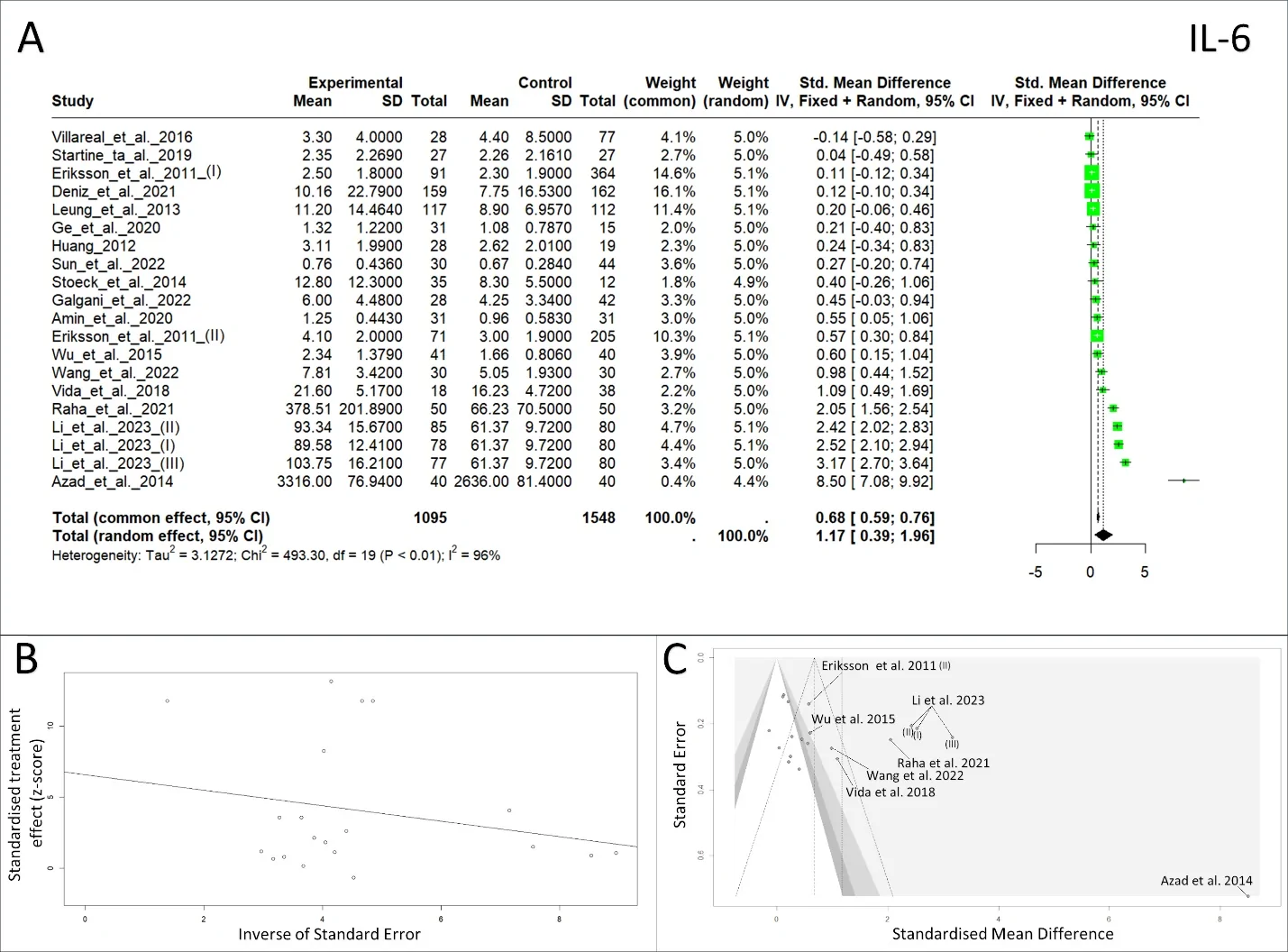
**Results for IL-6 in AD**. Forrest plot with total effect and the confidence interval for each study (A) Egger test for publication bias (B) and funnel plot showing studies which have significant effect sizes (C) for IL-6; in funnel plot, shades of gray indicate the confidence interval (0.99, 0.95, and 0.90)

*IL-6. S*eventeen studies with *n* = 1095 participants constituting the study group diagnosed with Alzheimer's disease and *n* = 1548 controls were analyzed. The overall concentration of IL-6 was found to be higher in the AD group compared to controls and the difference was significant, with a *p*-value of 0.0034 (SMD,1.17; 95% CI, 0.39-1.96). The heterogeneity was considerable with Q = 493.3 (*p*<0.01) and I^2^ =96% (Fig. 2A). Egger’s test for the publication bias was also found to be significant (*p*=0.0242) (Fig. 2B) and the asymmetry of the funnel plot was observed (Fig. 2C). However, after removing the biased dataset, the difference between control and patient group remains significant.

**Figure 3.**
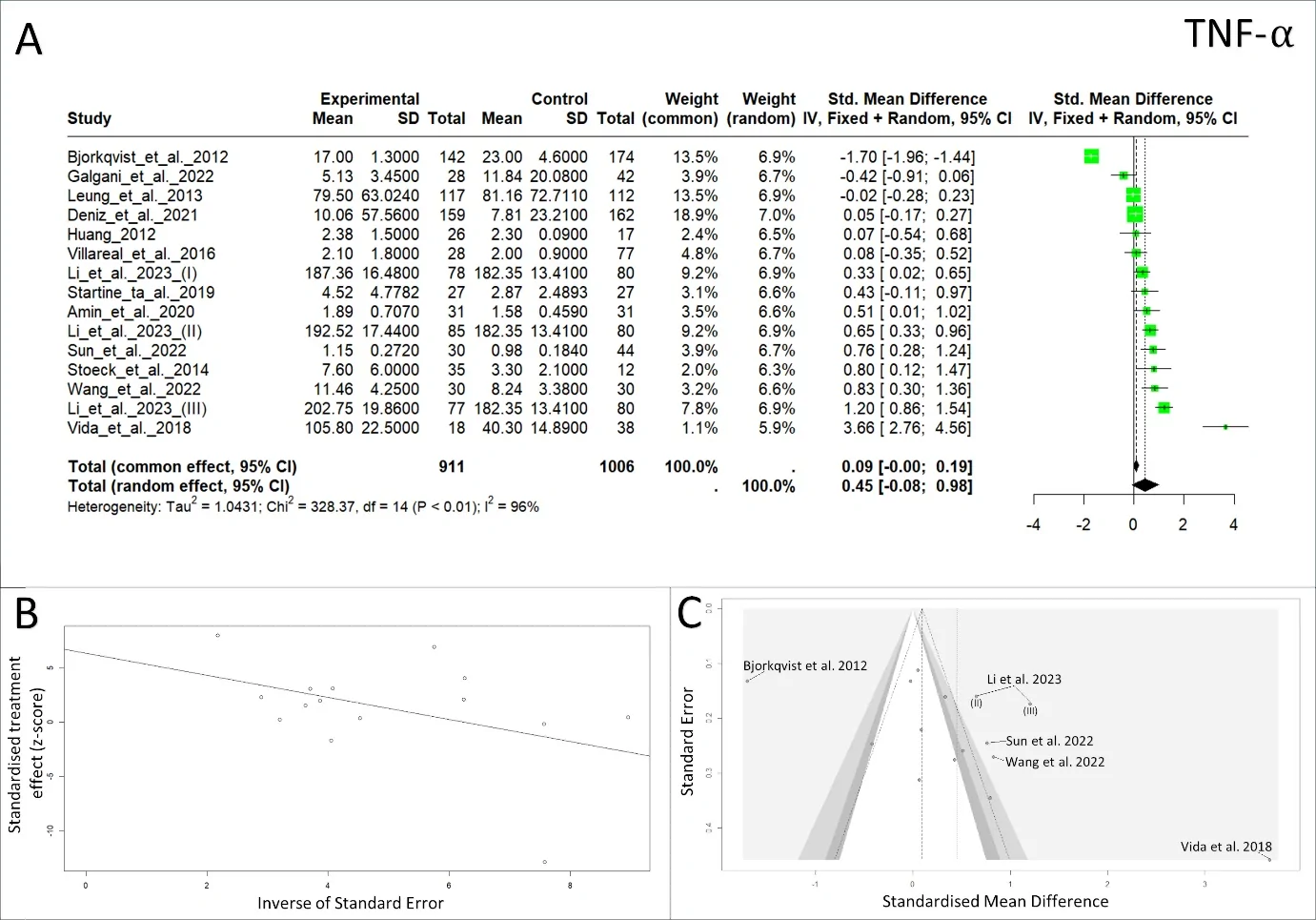
**Results for TNF-α in AD**. Forrest plot with total effect and the confidence interval for each study (A) Egger test for publication bias (B) and funnel plot showing studies which have significant effect sizes (C) for TNF-α; in funnel plot, shades of gray indicate the confidence interval (0.99, 0.95, and 0.90)

*TNF-α.* The AD group for TNF-α analysis involved *n*=911 patients and the number of controls reached *n*=1006. The random effect for this cytokine was SMD 0.45, 95% Cl -0.08-0.98 with *p*-value =0.9882, suggesting that TNF-α values were not significantly different in the AD group when compared to the control group. The observed heterogeneity of the reported studies was high (Q =328.37; P < 0.01; I^2^ = 96%) (Fig. 3A). Egger’s test for the publication was not significant *(p* =0.0656) (Fig. 3B), and the asymmetry of the funnel plot was not observed (Fig. 3C).

**Figure 4.**
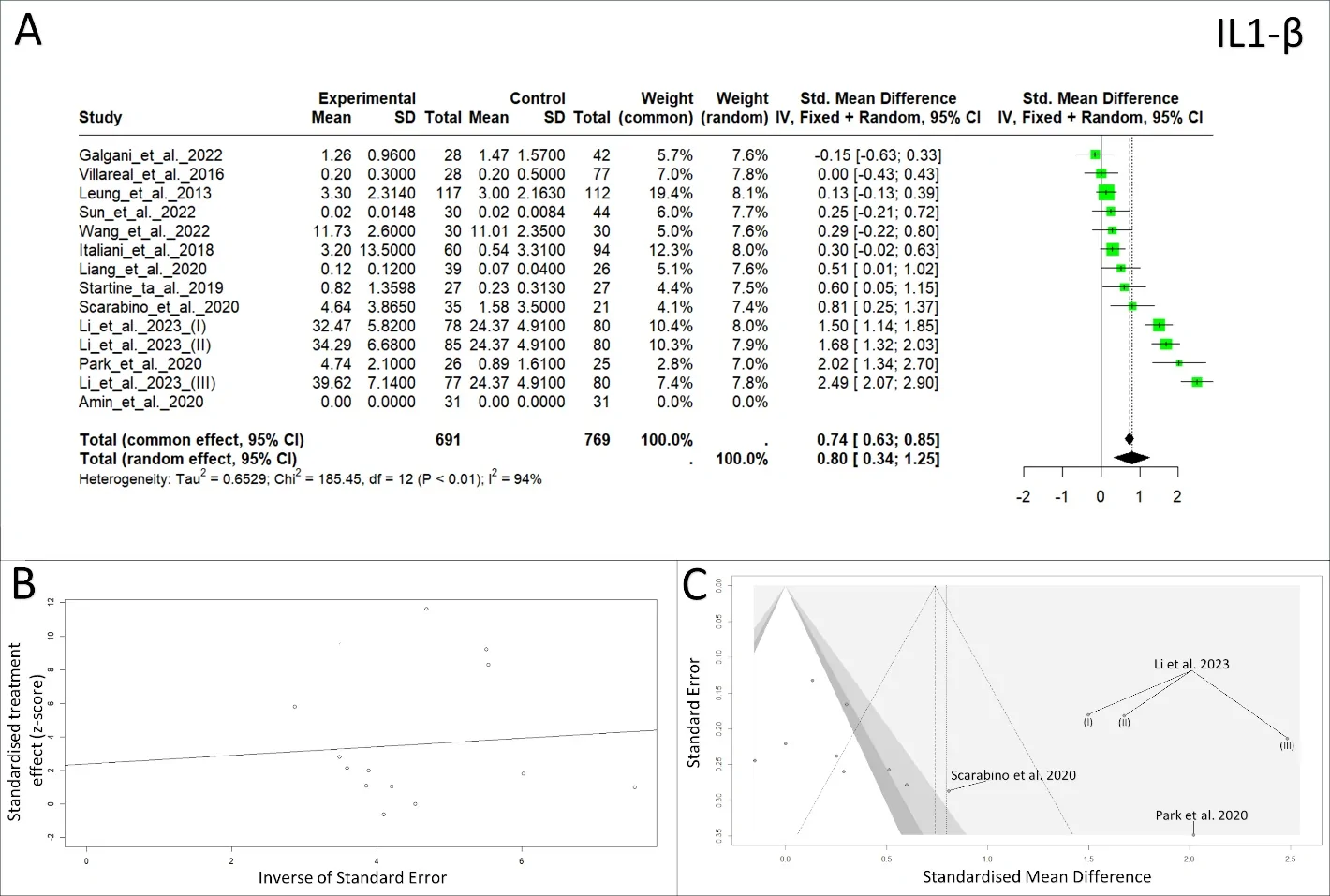
**Results for IL1β in AD**. Forrest plot with total effect and the confidence interval for each study (A) Egger test for publication bias (B) and funnel plot showing studies which have significant effect sizes (C) for IL-1β; in funnel plot, shades of gray indicates the confidence interval (0.99, 0.95, and 0.90)

*IL-1β.* In the assessment of the role of IL-1β in the pathogenesis of AD, 14 records (12 publications) were included, in which the number of AD patients reached *n*=691 individuals and the control group consisted of *n*=761. The results of the analysis showed a random effect value of SMD 0.80 95% Cl 0.34-1.25, and *p*-value 0.0006, which indicates a significantly higher level of IL-1β in the patients group. The heterogeneity of the test was Q =185.45; *p* < 0.01; I^2^ = 94% (Fig. 4A). Egger’s test for the publication showed *p* =0.5989 (Fig. 4B), and the asymmetry of the funnel plot was not significant (Fig. 4C).

*IL-8, IL-12, IL-18 and IFN-γ.* The remaining pro-inflammatory cytokines assessed did not show any significance in the random effect. Nevertheless, under the common effect model, three cytokines showed significant *p*-values: IL-8, IL-18 and IFN-γ (Table 4). The results obtained under the common effect model (SMD -0.3967 95% Cl -0.5417-0.3517 *p*-value=0.0001) may indicate a higher level of IL-8 in the control group.

**Table 4:** Results of the heterogeneity tests

Factor	Disease	N	Random effects model				Common effects model			
			SMD	95%-CI	z	p-value	SMD	95%-CI	z	p-value
**Pro-inflammatory cytokines**										
**IL-8**	AD	6	-0.0566	[-0.5896; 0.4763]	-0.2	0.835	-0.3967	[-0.5417; -0.2517]	-5.36	< 0.0001
**IL-12**	AD	3	0.0661	[-0.1411; 0.2733]	0.63	0.5318	0.0661	[-0.1411; 0.2733]	0.63	0.5318
**IL-18**	AD	4	0.5007	[-0.3861; 1.3876]	1.11	0.2684	0.3258	[ 0.1263; 0.5253]	3.2	0.0014
**IFN-γ**	AD	3	2.7761	[-1.8811; 7.4334]	1.17	0.2427	0.4675	[ 0.2395; 0.6955]	4.02	< 0.0001
**Anti-inflammatory cytokines**										
**IL-4**	AD	3	-0.2815	[-1.0578; 0.4948]	-0.7	0.4772	-0.1963	[-0.4030; 0.0104]	-1.86	0.0627
**IL-10**	AD	6	0.1493	[-0.0058; 0.3044]	1.89	0.0593	0.1436	[0.0059; 0.2813]	2.04	0.041
**IL-13**	AD	4	0.073	[-0.1296; 0.2755]	0.71	0.48	0.073	[-0.1296; 0.2755]	0.71	0.48

Alzheimer’s disease (AD); standardized mean difference (SMD); confidence interval (CI); interleukin 8 (IL-8); interleukin 12 (IL-12); interleukin 18 (IL-18); interferon γ (IFN-γ); interleukin 4 (IL-4); interleukin 10 (IL-10); interleukin 13 (IL-13)

### Anti-inflammatory cytokines

Three anti-inflammatory cytokines: IL-4, IL-10 and IL-13, showed no significance in the random effect. The common effect for IL-10 was SMD 0.1436 95% Cl 0.0059; 0.2813, *p*=0.041, which indicates that this cytokine values were higher in the AD group compared to the control group and may suggest its diagnostic usefulness. No differences in common effects were detected for IL-4 and IL-13.

### Assessment of proinflammatory and anti-inflammatory cytokines in Parkinson’s disease

#### Proinflammatory cytokines

**Figure 5.**
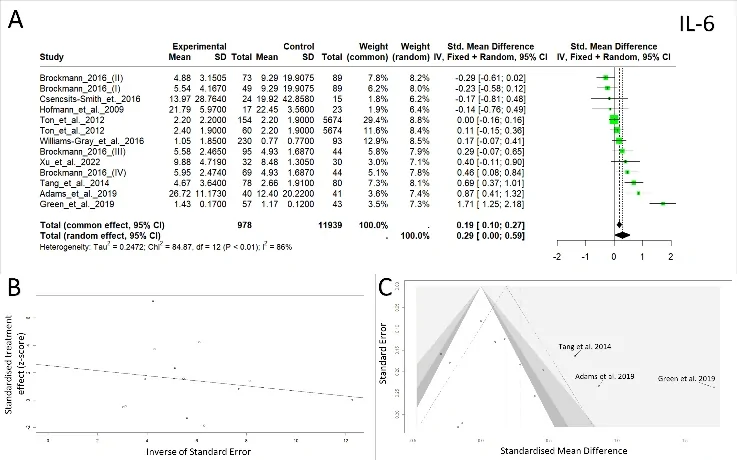
**Results for IL-6 in PD**. Forrest plot with total effect and the confidence interval for each study (A) Egger test for publication bias (B) and funnel plot showing studies which have significant effect sizes (C) for IL-6; in funnel plot, shades of gray indicates the confidence interval (0.99, 0.95, and 0.90)

*IL-6.* Nine publications assessed the role of IL-6 in the pathogenesis of PD. In total, there were 978 individuals in the PD group and *n* = 11,939 people in the control group. The random effect analysis showed statistical significance at the level of *p*-value = 0.0487, SMD 0.29 95% Cl 0.00-0.59. Heterogeneity was significant (Q =84.87; P < 0.01; I^2^ = 96%) (Fig. 5A). Egger’s test for the publication showed *p* =0.204 (Fig. 5B), and the asymmetry of the funnel plot was not observed (Fig. 5C).

**Figure 6.**
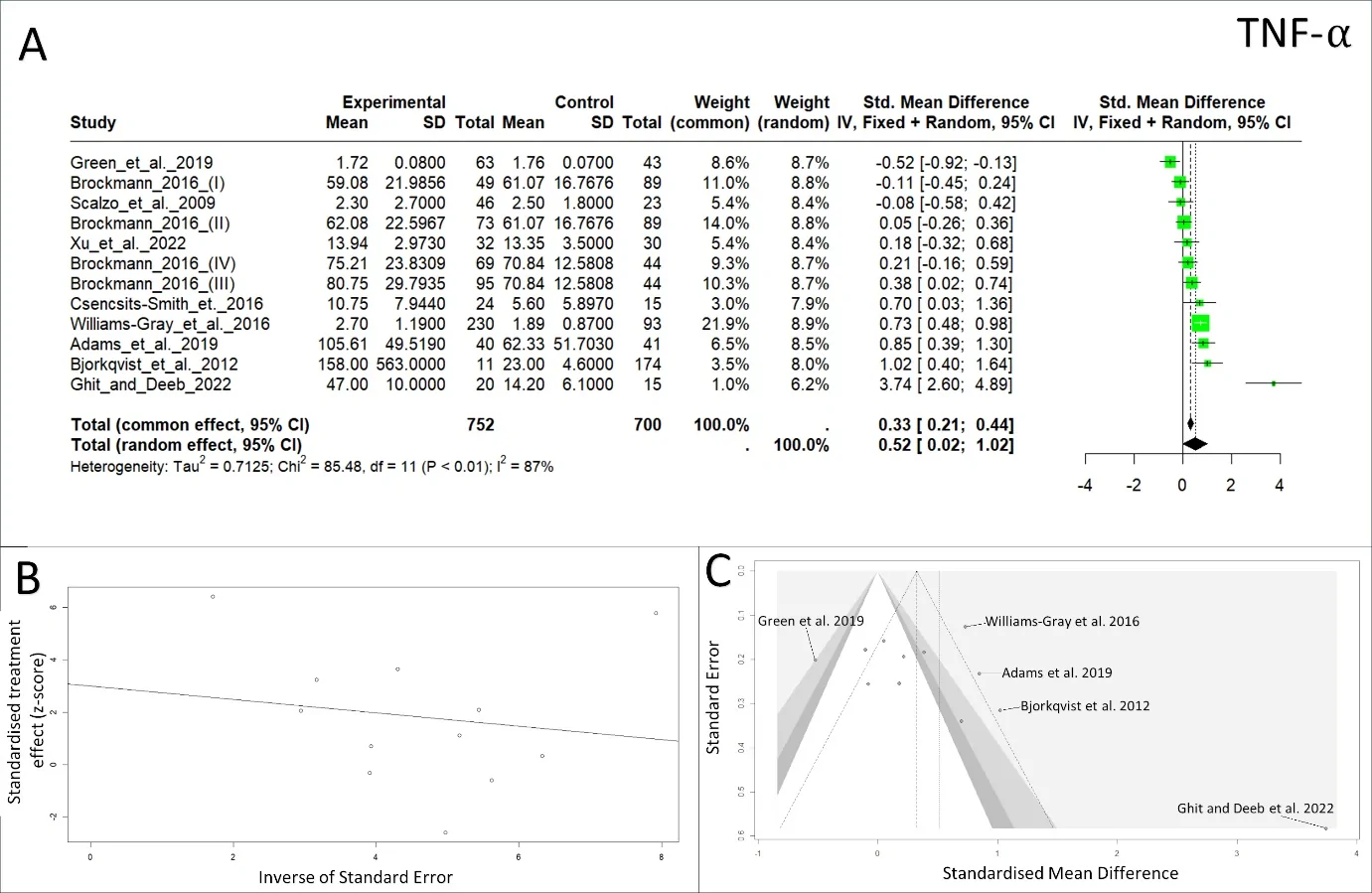
**Results for TNF-α in PD**. Forrest plot with total effect and the confidence interval for each study (A) Egger test for publication bias (B) and funnel plot showing studies which have significant effect sizes (C) for TNF-α; in funnel plot, shades of gray indicates the confidence interval (0.99, 0.95, and 0.90)

*TNF-α.* Random-effects results demonstrated that patients with PD (*n*=752) had a higher level of TNF-α compared to the control group (*n*=700). Statistical significance was at *p*= 0.0431, SMD 0.52 95%Cl 0.02-1.02. Heterogeneity was significant (Q =85.48; P < 0.01; I^2^ = 87%) (Fig. 6A). Egger’s test was at the level of *p*-value =0.2435 (Fig. 6B), and the asymmetry of the funnel plot was not observed (Fig. 6C).

**Figure 7.**
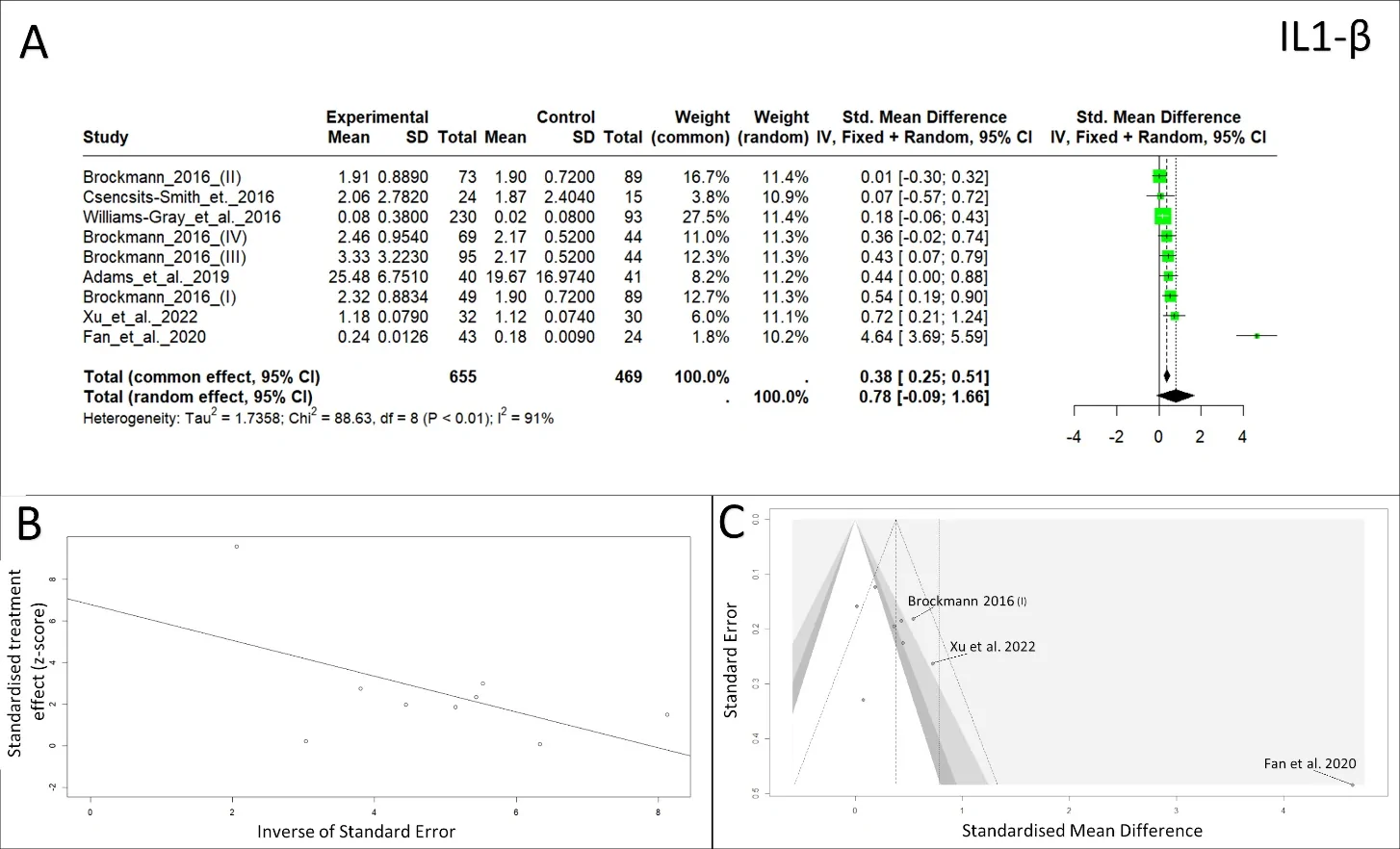
**Results for IL-1β in PD**. Forrest plot with total effect and the confidence interval for each study (A) Egger test for publication bias (B) and funnel plot showing studies which have significant effect sizes (C) for IL-1β; in funnel plot, shades of gray indicate the confidence interval (0.99, 0.95, and 0.90)

*IL-1β*. In IL-1β assessment, the group of patients with PD was larger *(n*=655) than the control group (*n*=469). The total number of records assessed was nine (6 publications). Heterogeneity was high (Q =88.63; p< 0.01; I^2^ = 91%) (Fig. 7A). Egger’s test for the publication has *p* = 0.0313 (Figure 7B), and the asymmetry of the funnel plot was observed (Fig. 7C). After removing the biased dataset, the levels of cytokines start to be significantly higher in the patient group (Std. mean difference for the common effect 0.30 [0.17; 0.43], for the random effect 0.32 [0.16; 0.48]).

*IL-8, IL-12, IL-18 and IFN-γ.* There were no differences between IL-8, -12, -18 and IFN-*γ* between groups in the random effect model. In turn, the common effect showed significance for IL-8 (SMD 0.154 95% Cl 0.0227-0.2854, *p*-value = 0.0216) and IL-12 (SMD 0.4865 95% Cl 0.0855-0.8874 *p*-value =0.0174), which could indicate higher values of these cytokines in patients’ group (Table 5.).

#### Anti-inflammatory cytokines

There were no differences in the random effect for the following cytokines: IL-4, IL-10, and IL-13. Statistically significant differences in common effect (SMD 0.3107 95% Cl 0.1849; 0.4365, *p*-value=0.0001) were observed for IL-10. This could be indicative of higher values of this cytokine in PD patients in the studies included in our meta-analysis (Table 5).

**Table 5:** Results of the heterogeneity tests.

Factor	Disease	N	Random effects model				Common effects model			
			SMD	95%-CI	z	p-value	SMD	95%-CI	z	p-value
**Pro-inflammatory cytokines**										
**IL-8**	PD	7	0.1927	[-0.0584; 0.4438]	1.5	0.1325	0.154	[ 0.0227; 0.2854]	2.3	0.0216
**IL-12**	PD	3	1.8106	[-1.5748; 5.1961]	1.05	0.2945	0.4865	[ 0.0855; 0.8874]	2.38	0.0174
**IL-18**	PD	4	-0.1575	[-0.3632; 0.0481]	-1.5	0.1333	-0.1636	[-0.3370; 0.0098]	-1.85	0.0645
**IFN-γ**	PD	3	0.0964	[-0.1042; 0.2970]	0.94	0.3461	0.0964	[-0.1042; 0.2970]	0.94	0.3461
**Anti-inflammatory cytokines**										
**IL-4**	PD	7	0.0772	[-0.1017; 0.2561]	0.85	0.3979	0.0753	[-0.0560; 0.2065]	1.12	0.2609
**IL-10**	PD	9	0.6667	[-0.1403; 1.4737]	1.62	0.1054	0.3107	[ 0.1849; 0.4365]	4.84	< 0.0001
**IL-13**	PD	3	0.0709	[-0.1479; 0.2897]	0.63	0.5254	0.0603	[-0.1403; 0.2609]	0.59	0.5559

Parkinson’s disease (PD); standardized mean difference (SMD); confidence interval (CI); interleukin 8 (IL-8); interleukin 12 (IL-12); interleukin 18 (IL-18); interferon γ (IFN-γ); interleukin 4 (IL-4); interleukin 10 (IL-10); interleukin 13 (IL-13)

## DISCUSSION

The characteristic neuropathological features in the development and pathogenesis of AD include 1) extracellular Aβ plaques and 2) intracellular hyperphosphorylated tau (p-τ) in neurofibrillary tangles [79,80]. A number of studies point to the multifactorial etiology of AD [81,82] including the inflammation of the nervous system as one of the pathological hallmarks of the disease, which may exacerbate Aβ and τ pathology [83,84]. Experimental studies have shown that Aβ deposition in the brain can cause brain inflammation, which results in an increased release of pro-inflammatory cytokines [85]. The report by von Bernhard et al. [86] indicated that the binding of Aβ to the surface of microglia could activate pro-inflammatory genes and increase the amount of pro-inflammatory cytokines such as TNF-α, IL-1β, IL-6, IL-8 and IL-18, which leads to tau hyperphosphorylation and a significant neuronal loss. In the available studies, particular attention has been paid to two cytokines, IL-6 and IL-8, which do not only increase with age but are also associated with Aβ accumulation. Surprising observations were reported by Hesse et al. [23] where IL-8 levels in CSF were found to be lower in AD patients than in controls (AD: 35.0 / 29.67-46.16 pg/ml, control: 41.73/36.73-58.74 pg/ml; *p*=0.02), which is inconsistent with our observations in this meta-analysis where no differences were detected between AD patients vs. controls (SMD 0.1927 95 % Cl [-0.0584; 0.4438] *p*=0.1325). Nonetheless, numerous experimental studies have demonstrated a considerable role of IL-6 in neuroinflammation, e.g. Cojocaru et al. [87] recorded significantly elevated Il-6 levels in AD patients (234 pg/ml, range 85-567 pg/ml) when compared to the control group (67 pg/ml, range 38-181 pg/ml); *p*< 0.001. Our analysis which included *n*=17 publications (twenty research papers), revealed a statistically significant relationship between AD patients and the control group in the level of IL-6 (*p*-value=0.0034 (SMD,1.17; 95% CI, 0.39-1.96). The results of our observations are inconsistent with the findings of the meta-analysis by Ng et al. [88], where *n*=8 studies were included and no significant relationship between AD and controls was identified (random-effects model: 0.228, 95% CI: -0.074-0.528, z=1.482, *p*=0.138). By contrast, consistent observations were made by Swardfager et al. [89] who detected significantly higher peripheral blood concentrations of IL-6 in the AD group than in the control group (2.86 95% Cl 1.68, 4.04, *p*< 0.00001). It is worth emphasizing that IL-6 as a pleiotropic cytokine can act both neuroprotectively and neurodegeneratively depending on the initial state of the neurons in patients. It has been shown that initial IL-6 stimulation has a destructive effect, but subsequent progressive neurodegeneration can lead to the neuroprotective effect of IL-6 [90]. Wang et al. [85] mentioned that IL-6 levels increase mainly as a result of plaque formation in the early stages of the disease. Therefore, it seems obvious that the evaluation of this cytokine and its exact role in the pathogenesis of AD requires further studies taking into account the stage of the patient's disease, as well as sex. Studies conducted on mouse models demonstrated that central overexpression of IL-6 could lead to the expression of TNF-α and microglial cells, which may impair cognitive and metabolic functions [85]. Paganelli et al. [91] recorded lower levels of TNF-α in mild and moderate AD compared to the severe AD stage, which may suggest stage-dependent levels of this cytokine. Probert [92] also sugget tht TNF-α is strongly associated with cognitive impairment in both mild and severe AD. In the publications included in our meta-analysis, the lowest TNF-α values were reported by Amin et al. [47], where approximately 40% of the subjects were women, while the highest TNF-α values were recorded by Li et al. [45] in the severe AD group (202.75±19.86 pg/ml), where women constituted 60% of the subjects. Although there is no clear evidence of differences in the incidence of the disease between the sexes. Women and men also exhibit different clinical features in terms of the duration of AD and associated neuropsychiatric disorders. Women with AD have a more rapid progression of hippocampal atrophy and a higher burden of AD pathology (i.e., amyloid plaques and neurofibrillary tangles) compared to men [93]. Furthermore, women have also been shown to exhibit a significant increase in inflammation around menopause. Furthermore, female has been associated with greater expression of inflammation-related genes in the hippocampus with age [94]. The Framingham study, which included *n*=1,550 women and *n* =1,061 men observed for over 20 years, showed that the lifetime risk of the disease for 65-year-old men was 6.3 %, while it was twice as high and amounted to 12.1% in women [95]. Despite these relationships and disproportions between sexes, no significance between AD and the control group was noted in our meta-analysis (SMD 0.45, 95% Cl -0.08-0.98 with *p*-value=0.9882). Increased serum IL-1β levels are used as an indicator of progressive neurodegeneration in the brain [96] and elevated IL-1β obviously promotes the disease progression [97]. Research conducted by Torres et al. [98] showed a significant increase in CD14+ monocytes expressing IL-1β in AD patients compared to controls. Both Liang et al. [57] and Amin et al. [47] also demonstrated statistically significantly higher IL-1β values in AD patients compared to the control group. Over the years, the role of this cytokine has been assessed and in 2010 a meta-analysis by Swardfager et al. [89] reported a significant relationship between Alzheimer's disease and the control group (95% Cl 0.55 [0.32, 0.78] pg/mL, *p*< 0.00001, *n*= 574 AD patients/*n*=370 control patients, 10 studies). Moreover, similar observations were made in 2018 by Ng et al. [88], where significantly higher peripheral blood IL-1β levels were identified in elderly patients with Alzheimer's disease than in control participants (SMD: 1.37, 95% CI: 0.06-2.68, z=1.98, *p*=0.041). Our meta-analysis also indicated a role of IL-1β in neuroinflammation in AD patients (SMD 0.80 95% Cl 0.34-1.25, and *p*-value 0.0006). Other anti-inflammatory cytokines assessed in our meta-analysis did not show any significance in the random effect. Nevertheless, the significance of the common effect (SMD 0.1436 95% Cl [0.0059; 0.2813] *p*-value =0.041) was recorded for IL-10, which indicates higher values of this cytokine in AD patients. Studies on animal models revealed that IL-10 overexpression in the hippocampus of AD mice could enhance neurogenesis and improve cognitive function, which can potentially be explained by the neuroprotective role of this cytokine in this pathological condition [21,96].

Chronic inflammation destroys the blood-brain barrier and activates microglia, which leads to neuroinflammation, and degeneration of dopaminergic neurons and, consequently, promotes the development of Parkinson's disease [94]. Excessive activation of microglia is associated with an increased release of pro-inflammatory cytokines: TNF-α [100], IL-1β [101], IL-2 [102], IL-6 [103] thus becoming a contributing factor in neuropathological processes in PD [102]. Sex is a recognized risk factor for Parkinson's disease, with a male to female ratio is 3:2. In women, the incidence of the disease is lower in the 50-59 age group [104]. Interestingly, in the case of PD, ethnicity is also a risk factor, with Hispanics having the highest incidence of PD (16.6, 95% CI: 12.0, 21.3) in the United States, followed by Blacks (10.2, 95% CI: 6.4, 14.0), Asians (11.3, 95% CI: 7.2, 15.3), and non-Hispanic Whites (13.6, 95% CI: 11.5, 15.7) [105]. Research conducted by Scalzo et al. [106] on a group of *n*=44 patients with PD and *n*=22 healthy controls reported statistically significantly higher IL-6 levels in the former. Patients with PD need significantly more time to perform functional mobility tests and their walking speed is slower in comparison to healthy controls Thirteen studies (9 publications) included in our meta-analysis assessed the role of IL-6 in the pathogenesis of PD. In total, there were *n*= 978 individuals in the PD group and *n* =11,939 people in the control group. The random effect analysis showed statistical significance at the level of *p*-value = 0.0487, SMD 0.29 95% Cl 0.00-0.59. Hofmann et al. [75] reported a negative correlation between IL-6 levels and activities of daily living (*p*<0.05), which emphasizes the fact that inflammatory mechanisms may be involved in neurodegenerative processes in PD. In patients with Parkinson's disease, higher levels of pro-inflammatory cytokines were found to be associated with poorer cognitive performance [107], and research by Lindqvist et al. [29] indicated that plasma TNF-α levels were positively correlated with cognitive impairment, but also increased the risk of depression. Recent research by Diaz et al. [108] on patients with a mean age of 72.76 ± 7.14 (*n*=26 patients with PD, *n*=14 healthy controls) demonstrated statistically significantly higher TNF-α values in the PD group compared to controls (*p*>0.05). Our meta-analysis of 8 publications, including the study by Brockmann et al. [70] which was assessed 4 times, showed a significant relationship with higher TNF-α values were detected in PD patients. These observations are similar to the reports in the meta-analysis by Qin et al. [109] (Hedges g, 0.354; 95% CI, 0.144-0.563; *p* = 0.001), and also by Qu et al. [110] (Hedges’ g 0.593; 95%CI 0.293 to 0.894, *p*<0.001). In contrast to the significance for IL-6 and TNF-α observed in our meta-analysis, no between-group significance was identified for IL-1β. The lack of significance may be attributed to the fact that the results reported in the two included publications i.e. Brockmann et al. [70] and Csencsits-Smith et al. [72], showed similar levels between the control group and patients with PD were very similar. These observations depart from the findings reported by Ferrari et al. [111] where long-term expression of IL-1β was observed to cause a marked loss of dopaminergic neurons and motor symptoms of PD. Moreover, a large-center study conducted by Williams-Gray et al. [32] also indicated higher IL-1β values in PD patients (*p* ≤ 0.001). However, the exact role of IL-12 or IL-8 in the pathogenesis of PD is yet to be clearly determined. In our meta-analysis, we assessed both IL-12 (*n*=3 publications) and IL-8 (*n*=4 publications) and did not observe any significance in the random effect. Diaz et al. [108] did not report any significance between the PD patient and the control group, although the levels of both cytokines were higher in the PD group. These observations require inclusion in subsequent meta-analyses of further publications that will focus on the assessment of IL-8 or IL-12 concentrations in PD. In turn, as in Alzheimer's disease patients, it has been shown that, unlike IL-6, IL-10 appears to have a beneficial effect on PD progression [112]. Studies on a lipopolysaccharide-induced mouse model of PD indicated that intracerebral IL-10 reduced the number of activated microglia and inhibited TNF-α and nitric oxide production [76]. Although our literature review and meta-analysis revealed some significance only in the common effect (SMD 0.3107 95%Cl [0.1849; 0.4365] *p*< 0.0001), not in the random effect model, further investigation of the in-depth role of this cytokine in neuroinflammation associated with PD is warranted in future research. In order to thoroughly investigate the relationship between inflammation and the role of cytokines in the pathogenesis of Alzheimer's and Parkinson's disease, longitudinal studies are needed. Assessment of cytokine levels over time in the same individuals will allow us to understand the dynamics of neuroinflammation, which may result in more targeted diagnostics and, consequently, therapy.

Although the presented study is a robust summary of a set of research papers, several limitations should be acknowledged. Firstly, we focused only on the assessment of cytokine levels determined in the blood. Secondly, the patients were of different ages and sexes, which may also make the interpretation of the results difficult. Additionally, our meta-analysis included only articles from two databases which meet certain criteria, like full English text; thus, some of the existing and relevant studies may have been overlooked. Although in most cases some of the publications were visibly different from the rest (Fig. 2-7), a significant bias was detected only for IL-6 in Alzheimer's disease and for IL-1b in Parkinson's disease. When the outlier [48] was removed, it had no impact on the results of the former, however, in the latter, the outlier removal [66] affected the results, and IL-1b began to reach significantly higher levels in the patient group.

## Conclusion

Our meta-analysis revealed elevated blood IL-6 values in both Alzheimer's and Parkinson's disease patients. In contrast, the role of TNF-α was demonstrated exclusively in PD whereas IL-1β was found to be significant in AD patients. The body of literature supporting this review and meta-analysis reinforces the clinical evidence of the presence of a peripheral inflammatory response in patients with neurodegenerative diseases, including Alzheimer's and Parkinson's disease.

## Supplementary Materials

The Supplementary data can be found online at: www.aginganddisease.org/EN/10.14336/AD.2024.1174.

## Data Availability

The raw data necessary to perform the analysis are available from the corresponding author on reasonable request.
