# Supplementary material for: Neuroinflammation as a Link in Parkinson’s and Alzheimer’s Diseases: A Systematic Review and Meta-Analysis
Source: Aging Dis. 2024 Dec 7;16(6):3584–600. doi: 10.14336/AD.2024.1174 (PMC12539528; doi:10.14336/AD.2024.1174)
Supplement: Supplementary file 1 — The Supplementary data can be found online at: www.aginganddisease.org/EN/10.14336/AD.2024.1174. [file AD-16-6-3584-s.pdf]

SUPPLEMENTARY DATA

Neuroinflammation as a Link in Parkinson's and Alzheimer's Diseases: A Systematic Review and Meta-Analysis

Anna Tylutka, Piotr Żabiński, Łukasz Walas, Agnieszka Zembron-Lacny

© 2024. Tylutka A. et al. Published online at http://www.aginganddisease.org/EN/10.14336/AD.2024.1174

SUPPLEMENTARY DATA

Supplementary Table 1. Values for AD.

Study	Disease					Control				
	N	Age (years)	% of women	Mean	SD	N	Age (years)	% of women	Mean	SD
**Pro - inflammatory**										
**IL-6 (pg/ml)**										
Deniz et al. 2021	159	78.2±8.97	69.81	10.16	22.79	162	82.7±8.15	76.54	7.75	16.53
Galgani et al. 2022	28	73.1±5.3	57	6.00	4.48	42	72.3±4.7	60	4.25	3.34
Stoeck et al. 2014	35	69.5 ±2.5	57.1	12.80	12.30	12	62.5 ±2.5	50	8.30	5.50
Leung et al. 2013	117	76.2 ±6.09	66.66	11.20	14.46	112	72.3 ±6.72	46.4	8.90	6.96
Villareal et al. 2016	28	81.9 (9.2)	78.6	3.30	4.00	77	76.5 ±6.7	64.9	4.40	8.50
Huang 2012	28	83.00 ±6.8	14.3	3.11	1.99	19	79.89 ±7.0	26.3	2.62	2.01
Eriksson et al. 2011 (I)	91	79.5 ±6.2	68.1	2.50	1.80	364	78.2 ±6.6	60.2	2.30	1.90
Eriksson et al. 2011 (II)	71	81.6 ±5.3	73.2	4.10	2.00	205	81.3 ±5.6	62	3.00	1.90
Amin et al. 2020	31	74.1±7.4	41.9	1.25	0.44	31	66.0±8.6	54.8	0.96	0.58
Azad et al. 2014	40	74±7.49	60	3316.00	76.94	40	71.5±7.72	60	2636.00	81.40
Startine ta al. 2019	27	59.33 ± 4.04	33.3	2.35	2.27	27	49.26 ± 10.40	40.7	2.26	2.16
Ge et al. 2020	31	68.58 ±8.04	74.2	1.32	1.22	15	64.80 ±6.00	66.7	1.08	0.79
Li et al. 2023 (I)	78	69.36 ± 5.24	55.1	89.58	12.41	80	75.36 ± 9.32	53.8	61.37	9.72
Li et al. 2023 (II)	85	74.54 ± 6.16	51.8	93.34	15.67	80	75.36 ± 9.32	53.8	61.37	9.72
Li et al. 2023 (III)	77	76.76 ± 6.25	59.7	103.75	16.21	80	75.36 ± 9.32	53.8	61.37	9.72
Wang et al. 2022	30	79.8± 8.55	20	7.81	3.42	30	78.23± 9.71	26.7	5.05	1.93
Raha et al. 2021	50	72± 23	40	378.51	201.89	50	50± 27	60	66.23	70.50
Wu et al. 2015	41	73.1 ± 9.4	65.8	2.34	1.38	40	63± 5.6	67.5	1.66	0.81
Sun et al. 2022	30	75.27 ± 7.67	40	0.76	0.44	44	71.86 ± 7.76	50	0.67	0.28
Vida et al. 2018	18	79.1 ± 6.53	72.2	21.60	5.17	38	74.34 ± 9.22	26.3	16.23	4.72
**TNF-α (pg/ml)**										
Leung et al. 2013	117	76.2 ±6.09	66.66	79.5	63.024	112	72.3 ±6.72	46.4	81.16	72.711
Villareal et al. 2016	28	81.9 (9.2)	78.6	2.1	1.8	77	76.5 ±6.7	64.9	2	0.9
Huang 2012	26	83.00 ±6.8	14.3	2.38	1.5	17	79.89 ±7.0	26.3	2.3	0.09
Amin et al. 2020	31	74.1±7.4	41.9	1.8948	0.707	31	66.0±8.6	54.8	1.5848	0.459
Startine ta al. 2019	27	59.33 ± 4.04	33.3	4.5234	4.7782	27	49.26 ± 10.40	40.7	2.8687	2.4893
Li et al. 2023 (I)	78	69.36 ± 5.24	55.1	187.36	16.48	80	75.36 ± 9.32	53.8	182.35	13.41
Li et al. 2023 (II)	85	74.54 ± 6.16	51.8	192.52	17.44	80	75.36 ± 9.32	53.8	182.35	13.41
Li et al. 2023 (III)	77	76.76 ± 6.25	59.7	202.75	19.86	80	75.36 ± 9.32	53.8	182.35	13.41
Wang et al. 2022	30	79.8± 8.55	20	11.46	4.25	30	78.23± 9.71	26.7	8.24	3.38
Sun et al. 2022	30	75.27 ± 7.67	40	1.1535	0.272	44	71.86 ± 7.76	50	0.9816	0.184

© 2024. Tylutka A. et al. Published online at http://www.aginganddisease.org/EN/10.14336/AD.2024.1174

SUPPLEMENTARY DATA

Vida et al. 2018	18	79.1 ± 6.53	72.2	105.8	22.5	38	74.34 ± 9.22	26.3	40.3	14.89
Deniz et al. 2021	159	78.2±8.97	69.81	10.06	57.56	162	82.7±8.15	76.54	7.81	23.21
Galgani et al. 2022	28	73.1±5.3	57	5.13	3.45	42	72.3±4.7	60	11.84	20.08
Stoeck et al. 2014	35	69.5 ±2.5	57.1	7.6	6	12	62.5 ±2.5	50	3.3	2.1
Bjorkqvist et al. 2012	142	76 (56–87)	28.2	17	1.3	174	74 (62–99)	67.2	23	4.6
**IL1 - β (pg/ml)**										
Wang et al. 2022	30	79.8± 8.55	20	11.73	2.6	30	78.23± 9.71	26.7	11.01	2.35
Liang et al. 2020	39	77.7 ± 8.5	74.4	0.12	0.12	26	68.7 ± 4.2	80.8	0.07	0.04
Amin et al. 2020	31	74.1±7.4	41.9	0	0	31	66.0±8.6	54.8	0	0
Startin et al. 2019	27	59.33 ± 4.04	33.3	0.825	1.3598	27	49.26 ± 10.40	40.7	0.2251	0.313
Li et al. 2023 (I)	78	69.36 ± 5.24	55.1	32.47	5.82	80	75.36 ± 9.32	53.8	24.37	4.91
Li et al. 2023 (II)	85	74.54 ± 6.16	51.8	34.29	6.68	80	75.36 ± 9.32	53.8	24.37	4.91
Li et al. 2023 (III)	77	76.76 ± 6.25	59.7	39.62	7.14	80	75.36 ± 9.32	53.8	24.37	4.91
Italiani et al. 2018	60	78.13±8.35	41.7	3.2	13.5	94	68.64±6.95	43.6	0.54	3.31
Park et al. 2020	26	75.54 ± 6.17	69.2	4.74	2.1	25	75.56 ± 6.29	44	0.89	1.61
Sun et al. 2022	30	75.27 ± 7.67	40	0.0185	0.0148	44	71.86 ± 7.76	50	0.0156	0.0084
Leung et al. 2013	117	76.2 ±6.09	66.66	3.3	2.314	112	72.3 ±6.72	46.4	3	2.163
Galgani et al. 2022	28	73.1±5.3	57	1.26	0.96	42	72.3±4.7	60	1.47	1.57
Villareal et al. 2016	28	81.9 (9.2)	78.6	0.2	0.3	77	76.5 ±6.7	64.9	0.2	0.5
Scarabino et al.2020	35	78.5 ±8.2	71.1	4.64	3.865	21	70.0 ±8.9	61.1	1.5809	3.5
**IL-18 (pg/ml)**										
Villareal et al. 2016	28	81.9 (9.2)	78.6	168.5	91.2	77	76.5 ±6.7	64.9	234.1	141.6
Reale et al. 2017	38	73.8±5.5	47.4	247.8	92.6	39	72.7±4.8	53.8	102.6	11.4
Italiani et al.2018	60	78.13±8.35	41.7	309.8	324.3	94	68.64±6.95	43.6	245.6	96
Wu et al. 2015	41	73.1 ± 9.4	65.8	250.6	141.8	40	63± 5.6	67.5	249.6	132
Scarabino et al.2020	20	78.5 ±8.2	71.1	91.628	103.0817	15	70.0 ±8.9	61.1	37.7102	74.1012
**IL-12 (pg/ml)**										
Galgani et al. 2022	28	73.1±5.3	57	127.45	72.34	42	72.3±4.7	60	121.12	58.8
Leung et al. 2013	117	76.2 ±6.09	66.66	18	46.4243	112	72.3 ±6.72	46.4	15.4	24.933
Amin_et_al._2020	31	74.1±7.4	41.9	0.2508	0.272	31	66.0±8.6	54.8	0.2456	0.2021
**IFN -γ (pg/ml)**										
Amin et al. 2020	31	74.1±7.4	41.9	6.4038	3.5207	31	66.0±8.6	54.8	4.1988	2.1606
Azad et al. 2014	40	74±7.49	60	386.2	22.46	40	71.5±7.72	60	243	13.95
Leung et al. 2013	117	76.2 ±6.09	66.66	202.2	367.3434	112	72.3 ±6.72	46.4	170.6	200.6948
**IL-8 (pg/ml)**										
Bjorkqvist et al. 2012	142	76 (56–87)	28.2	10	0.5	174	74 (62–99)	67.2	11	1
Leung et al. 2013	117	76.2 ±6.09	66.66	7.5	3.8201	112	72.3 ±6.72	46.4	8.3	5.795

© 2024. Tylutka A. et al. Published online at http://www.aginganddisease.org/EN/10.14336/AD.2024.1174

Amin et al. 2020	31	74.1±7.4	41.9	8.3344	4.2357	31	66.0±8.6	54.8	7.9951	2.9922
Alsadany et al. 2012	25	72.2 ±5.9	56	12.6	3.4	25	72.8 ±4.1	52	11.6	1.6
Wang et al. 2022	30	79.8± 8.55	20	15.98	4.38	30	78.23± 9.71	26.7	13.42	4.36
Sun et al. 2022	30	75.27 ± 7.67	40	1.9597	1.1054	44	71.86 ± 7.76	50	1.7975	0.7204
**Anti - inflammatory**										
**IL-4 (pg/ml)**										
Leung et al. 2013	117	76.2 ±6.09	66.66	2.9	1.6212	112	72.3 ±6.72	46.4	3	1.9441
Amin et al. 2020	31	74.1±7.4	41.9	0.1351	0.1321	31	66.0±8.6	54.8	0.0975	0.1391
Azad et al. 2014	40	74±7.49	60	7.5	3.9	40	71.5±7.72	60	10.6	1.06
**IL-10 (pg/ml)**										
Deniz et al. 2021	159	78.2±8.97	69.81	1.85	6.57	162	82.7±8.15	76.54	1.58	3.52
Galgani et al. 2022	28	73.1±5.3	57	1.68	1.91	42	72.3±4.7	60	1.1	0.82
Leung et al. 2013	117	76.2 ±6.09	66.66	12.1	9.5374	112	72.3 ±6.72	46.4	10	7.2191
Villareal et al. 2016	28	81.9 (9.2)	78.6	1.6	1.7	77	76.5 ±6.7	64.9	2.8	6
Amin et al. 2020	31	74.1±7.4	41.9	0.5345	0.342	31	66.0±8.6	54.8	0.4136	0.3653
Startin et al.2019	27	59.33 ± 4.04	33.3	1.7005	2.1261	27	49.26 ± 10.40	40.7	1.2499	1.0543
**IL-13 (pg/ml)**										
Stoeck et al. 2014	35	69.5 ±2.5	57.1	6.7	7	12	62.5 ±2.5	50	4.3	6
Leung et al. 2013	117	76.2 ±6.09	66.66	9	7.6813	112	72.3 ±6.72	46.4	8.9	11.1625
Amin et al. 2020	31	74.1±7.4	41.9	1.8137	1.7098	31	66.0±8.6	54.8	1.2462	1.8031
Erhardt et al. 2021	19	70 (66-74)	47.4	2.403	3.3553	35	65 (62-68.5)	68.6	2.8393	2.9157

Supplementary Table 1. Values for PD.

Study	Disease					Control				
	**N**	**Age (years)**	**% of women**	**Mean**	**SD**	**N**	**Age (years)**	**% of women**	**Mean**	**SD**
**Pro - inflammatory**										
**IL-6 (pg/ml)**										
Csencsits-Smith et. 2016	24	64.2 ±8.6	66.7	13.97	28.76	15	56.8 ±9.2	66.7	19.92	42.86
Brockmann 2016 (I)	49	68 (46–83)	100	5.54	4.17	89	57 (18–83)	100	9.29	19.91
Brockmann 2016 (II)	73	67 (35–89)	100	4.88	3.15	89	57 (18–83)	100	9.29	19.91
Brockmann 2016 (III)	95	69 (46–80)	0	5.58	2.47	44	58 (28–83)	0	4.93	1.69
Brockmann 2016 (IV)	69	65 (36–88)	0	5.95	2.47	44	58 (28–83)	0	4.93	1.69
Ton et al. 2012 (I)	154	65+	45.4	2.20	2.20	5674	65+	58.2	2.20	1.90
Ton et al. 2012 (II)	60	65+	38.3	2.40	1.90	5674	65+	57.8	2.20	1.90

© 2024. Tylutka A. et al. Published online at http://www.aginganddisease.org/EN/10.14336/AD.2024.1174

Green et al. 2019	57	69.9±8.1	48.5	1.43	0.17	43	68.2±7.1	46.7	1.17	0.12
Tang et al. 2014	78	76.3 ±5.0	23.1	4.67	3.64	80	75.4 ±4.4	26.3	2.66	1.91
Adams et al. 2019	40	66 (62.3-72)	37.5	26.72	11.17	41	59 (53.5-72)	63.4	12.40	20.22
Xu et al. 2022	32	61.22 ± 8.54	53.1	9.88	4.72	30	55.77 ± 12.89	56.7	8.48	1.31
Williams-Gray et al. 2016	230	66.4 ±9.5	38.3	1.05	1.85	93	68.0 ±8.0	47.3	0.77	0.77
Hofmann et al. 2009	17	65.3 ±13.1	58.8	21.79	5.97	23	60.0 ±7.8	65.2	22.45	3.56
**TNF-α (pg/ml)**										
Bjorkqvist et al. 2012	11	72 (62–81)	45.45	158	563	174	74 (62–99)	67.2	23	4.6
Scalzo et al. 2009	46	65.8±8.9	50	2.3	2.7	23	61.9±10.8	65.2	2.5	1.8
Csencsits-Smith et. 2016	24	64.2 ±8.6	66.7	10.7457	7.944	15	56.8 ±9.2	66.7	5.5956	5.897
Brockmann 2016 (I)	49	68 (46–83)	100	59.0819	21.9856	89	57 (18–83)	100	61.0674	16.7676
Brockmann 2016 (II)	73	67 (35–89)	100	62.0846	22.5967	89	57 (18–83)	100	61.0674	16.7676
Brockmann 2016 (III)	95	69 (46–80)	0	80.7522	29.7935	44	58 (28–83)	0	70.8367	12.5808
Brockmann 2016 (IV)	69	65 (36–88)	0	75.2112	23.8309	44	58 (28–83)	0	70.8367	12.5808
Green et al. 2019	63	69.9±8.1	48.5	1.72	0.08	43	68.2±7.1	46.7	1.76	0.07
Adams et al. 2019	40	66 (62.3-72)	37.5	105.6127	49.519	41	59 (53.5-72)	63.4	62.3294	51.703
Xu et al. 2022	32	61.22 ± 8.54	53.1	13.935	2.973	30	55.77 ± 12.89	56.7	13.35	3.5
Ghit and Deeb 2022	20	61.7 ± 14.6	45	47	10	15	61.8 ± 4.9	33.3	14.2	6.1
Williams-Gray et al. 2016	230	66.4 ±9.5	38.3	2.7	1.19	93	68.0 ±8.0	47.3	1.89	0.87
**IL1 - β pg/ml)**										
Csencsits-Smith et. 2016	24	64.2 ±8.6	66.7	2.0642	2.782	15	56.8 ±9.2	66.7	1.8672	2.404
Brockmann 2016 (I)	49	68 (46–83)	100	2.3248	0.8834	89	57 (18–83)	100	1.8994	0.72
Brockmann 2016 (II)	73	67 (35–89)	100	1.9101	0.889	89	57 (18–83)	100	1.8994	0.72
Brockmann 2016 (III)	95	69 (46–80)	0	3.3292	3.223	44	58 (28–83)	0	2.1681	0.52
Brockmann 2016 (IV)	69	65 (36–88)	0	2.4637	0.954	44	58 (28–83)	0	2.1681	0.52
Adams et al. 2019	40	66 (62.3-72)	37.5	25.4775	6.751	41	59 (53.5-72)	63.4	19.6706	16.974
Xu et al. 2022	32	61.22 ± 8.54	53.1	1.176	0.079	30	55.77 ± 12.89	56.7	1.12	0.074
Fan et al. 2020	43	58.40 ± 1.37	55.8	0.2373	0.0126	24	57.92 ± 1.58	54.2	0.1835	0.009
Williams-Gray et al. 2016	230	66.4 ±9.5	38.3	0.08	0.38	93	68.0 ±8.0	47.3	0.02	0.08
**IL1-8 (pg/ml)**										
Brockmann 2016 (I)	49	68 (46–83)	100	293.4564	89.2394	89	57 (18–83)	100	460.0674	655.2387
Brockmann 2016 (II)	73	67 (35–89)	100	293.3293	136.4604	89	57 (18–83)	100	460.0674	655.2387
Brockmann 2016 (III)	95	69 (46–80)	0	330.3728	158.8451	44	58 (28–83)	0	325.9203	123.0732
Brockmann 2016 (IV)	69	65 (36–88)	0	332.0424	134.9077	44	58 (28–83)	0	325.9203	123.0732
**IL-12 (pg/ml)**										
Ghit and Deeb 2022	20	64.2 ±8.6	66.7	9.3	1.7	15	56.8 ±9.2	66.7	2.1	0.3
Rentzos et al. 2009	41	61.7 ± 14.6	45	6.4995	6.1459	19	61.8 ± 4.9	33.3	6.0542	7.3689

© 2024. Tylutka A. et al. Published online at http://www.aginganddisease.org/EN/10.14336/AD.2024.1174

Csencsits-Smith et. 2016	24	67.5 ±8.1	51.2	4.0298	6.005	15	65.8 ±11.2	52.6	3.1618	3.746
**IFN -γ (pg/ml)**										
Csencsits-Smith et. 2016	24	64.2 ±8.6	66.7	13.6001	21.8292	15	56.8 ±9.2	66.7	17.0497	27.9147
Adams et. al. 2019	40	66 (62.3-72)	37.5	10.192	5.8131	41	59 (53.5-72)	63.4	8.3472	9.4724
Williams-Gray et al. 2016	230	66.4 ±9.5	38.3	14.43	61.21	93	68.0 ±8.0	47.3	9.79	18.02
**IL-8 (pg/ml)**										
Bjorkqvist et al. 2012	11	72 (62–81)	45.45	11	1.2	174	74 (62–99)	67.2	11	1
Brockmann 2016 (I)	49	68 (46–83)	100	11.6303	7.8839	89	57 (18–83)	100	14.0684	11.5379
Brockmann 2016 (II)	73	67 (35–89)	100	15.5279	12.0592	89	57 (18–83)	100	14.0684	11.5379
Brockmann 2016 (III)	95	69 (46–80)	0	54.415	154.8256	44	58 (28–83)	0	16.3135	17.0593
Brockmann 2016 (IV)	69	65 (36–88)	0	25.7091	43.5743	44	58 (28–83)	0	16.3135	17.0593
Adams et al. 2019	40	66 (62.3-72)	37.5	13.9705	12.6374	41	59 (53.5-72)	63.4	4.078	7.7854
Williams-Gray et al. 2016	230	66.4 ±9.5	38.3	11.11	11.14	93	68.0 ±8.0	47.3	10.43	12.12
**Anti - inflammatory**										
**IL-4 (pg/ml)**										
Csencsits-Smith et. 2016	24	64.2 ±8.6	66.7	14.9931	23.0901	15	56.8 ±9.2	66.7	22.6137	40.8129
Brockmann 2016 (I)	49	68 (46–83)	100	17.4927	5.5914	89	57 (18–83)	100	15.587	5.0405
Brockmann 2016 (II)	73	67 (35–89)	100	15.2636	4.4921	89	57 (18–83)	100	15.587	5.0405
Brockmann 2016 (III)	95	69 (46–80)	0	16.7458	7.2649	44	58 (28–83)	0	13.6521	5.0095
Brockmann 2016 (IV)	69	65 (36–88)	0	13.5672	4.7937	44	58 (28–83)	0	13.6521	5.0095
Adams et al. 2019	40	66 (62.3-72)	37.5	13.4299	17.424	41	59 (53.5-72)	63.4	15.6339	14.9922
Williams-Gray et al. 2016	230	66.4 ±9.5	38.3	0.02	0.04	93	68.0 ±8.0	47.3	0.02	0.08
**IL-10 (pg/ml)**										
Brockmann 2016 (I)	49	68 (46–83)	100	7.2075	7.6894	89	57 (18–83)	100	4.2947	4.0739
Brockmann 2016 (II)	73	67 (35–89)	100	4.6546	3.6515	89	57 (18–83)	100	4.2947	4.0739
Brockmann 2016 (III)	95	69 (46–80)	0	6.7903	6.9243	44	58 (28–83)	0	3.5303	1.3242
Brockmann 2016 (IV)	69	65 (36–88)	0	3.9342	2.5417	44	58 (28–83)	0	3.5303	1.3242
Adams et al. 2019	40	66 (62.3-72)	37.5	4.4402	2.3337	41	59 (53.5-72)	63.4	4.6391	4.8399
Xu et al. 2022	32	61.22 ± 8.54	53.1	8.861	1.613	30	55.77 ± 12.89	56.7	8.5	1.83
Ghit and Deeb 2022	20	61.7 ± 14.6	45	10.7	2.2	15	61.8 ± 4.9	33.3	2.5	0.3
Rentzos et al. 2009	41	67.5 ±8.1	51.2	8.3321	3.6107	19	65.8 ±11.2	52.6	6.9764	3.0437
Williams-Gray_et_al._2016	230	66.4 ±9.5	38.3	0.35	0.61	93	68.0 ±8.0	47.3	0.21	0.14
**IL-13 (pg/ml)**										
Csencsits-Smith et. 2016	24	64.2 ±8.6	66.7	20.7104	42.7127	15	56.8 ±9.2	66.7	9.0923	15.4582
Adams et al. 2019	40	66 (62.3-72)	37.5	3.6402	1.9185	41	59 (53.5-72)	63.4	2.9251	3.9411
Williams-Gray et al. 2016	230	66.4 ±9.5	38.3	0.5	0.71	93	68.0 ±8.0	47.3	0.52	0.81

© 2024. Tylutka A. et al. Published online at http://www.aginganddisease.org/EN/10.14336/AD.2024.1174
